# A canary, a coal mine, and imperfect data: determining the efficacy of open-source climate change models in detecting and predicting extreme weather events in Northern and Western Kenya

**DOI:** 10.1007/s10584-022-03444-6

**Published:** 2022-10-19

**Authors:** Alvin M. Igobwa, Jeremy Gachanja, Betsy Muriithi, John Olukuru, Angeline Wairegi, Isaac Rutenberg

**Affiliations:** 1 @iLabAfrica, Strathmore University, Student Center, 4th Floor Keri Road, Nairobi, Kenya; 2 Centre for Intellectual Property and Information Technology Law (CIPIT), Strathmore University, Thomas Moore Building, Nairobi West, Keri Road, Nairobi, Kenya

**Keywords:** Climate change, Food security, Extreme weather prediction, Agricultural insurance, Insurance-based index

## Abstract

Climate models, by accurately forecasting future weather events, can be a critical tool in developing countermeasures to reduce crop loss and decrease adverse effects on animal husbandry and fishing. In this paper, we investigate the efficacy of various regional versions of the climate models, RCMs, and the commonly available weather datasets in Kenya in predicting extreme weather patterns in northern and western Kenya. We identified two models that may be used to predict flood risks and potential drought events in these regions. The combination of artificial neural networks (ANNs) and weather station data was the most effective in predicting future drought occurrences in Turkana and Wajir with accuracies ranging from 78 to 90%. In the case of flood forecasting, isolation forests models using weather station data had the best overall performance. The above models and datasets may form the basis of an early warning system for use in Kenya’s agricultural sector.

## Introduction

Agriculture is vital to Kenya’s economy. It contributes 33% to the nation’s gross domestic product (GDP), employs more than 40% of the total population (70% of the rural population), accounts for 65% of the country’s export earnings, and is a driving force in the non-agricultural sectors as well (FAO in Kenya 2019). Unfortunately, the agricultural sector in Kenya is plagued by low and declining crop production and is particularly vulnerable to climate change disruptions due to its over-reliance on rain-fed agriculture (Kabubo-Mariara and Kabara 2018). The nation’s landscape is varied with arid and semi-arid zones (characterized by high temperatures and lands less suitable for arable agriculture) accounting for about 80% of the total land area (Kabubo-Mariara and Kabara 2018). In February 2022, Kenya’s Food Security Steering Group (KFSSG) reported that there are around 3.1 million food-insecure people in pastoral and marginal agricultural areas, a 48% increase since August 2021 (OCHA Services 2022). While food insecurity was a problem in Kenya before the advent of the COVID-19 pandemic, the pandemic has significantly food insecurity in the country. The issue of food security is part of Kenya’s government ‘big four’ agenda—the government’s strategic agenda for the time period between 2018 and 2022—in which reducing food insecurity is emphasized as an integral part of the country’s economic progress (Parliamentary Service Commission (PSC) (2018)). To mitigate food insecurity, Kenya’s government engages in a number of interventions including, but not limited to (Bahemuka 2017) the following:

i) Providing input, tools, and equipment to farmers;
ii) Providing mass livestock vaccinations, off-take program restocking and animal feeds;
iii) Providing fish stock, cold storage facilities, and fish equipment;
iv) Providing water tanks, borehole rehabilitation, distillation of water pans and dams;
v) Providing food relief; and.
vi) Providing health and nutrition packages.

The Kenyan government also supports efforts to increase agricultural productivity through development and application of technology. There are a number of ways in which technology may be integrated into the agricultural sector to increase production and reduce loss: in precision agriculture, as market linkages, for farmer financial inclusion, for use in early warning systems, and to enable data-driven decision-making by stakeholders in the agricultural sector. High penetration of mobile technology and the spread of digital infrastructure has revolutionized operations and communication within Kenya’s agricultural sector. There has been increased utilization of mobile platforms by the country’s farmers to solve various agricultural problems such as reducing information gaps facing farmers on variations in market prices and conditions, fluctuations in weather patterns, and knowledge on where to buy farm inputs (GeoPoll 2018). M-Shamba and MbeguChoice are examples of applications currently available to Kenyan farmers. M-Shamba provides subscribers with information on the weather and climate, production, credit, and harvesting; MbeguChoice provides farmers with information on the best crop varieties to use in their specific regions to boost crop yields, and MasterCard Farmer Network (MFN) platform previously known as 2kuze has incorporated mobile technology in linking farmers to the market (MShamba 2021; MbeguChoice 2022).

It is our contention that climate change models may be a tool utilized in the nation’s goal to reduce food insecurity. Climate models may be utilized in the agricultural sector as early warning systems to mitigate crop and animal loss from extreme weather phenomena. In this paper, we investigate the efficacy of various open-source climate change models and weather datasets in predicting possible drought and flood weather patterns in northern and western Kenya. In Section 1, we outline the geography and climates of the study areas of interest and give a brief overview of climate change models methods currently in use; Sections 2 and 3 outline the methodology and results of the study; and in Section 4, we discuss the results obtained and future work from this study.

### Extreme weather in Kenya

Kenya’s reliance on agriculture and other rain-fed activities to sustain its economy makes it particularly vulnerable to climate extremes. For example, recurring floods in areas along rivers flowing from Lake Victoria: Budalang’i in Busia County, Kano Plains in Kisumu County, and the lower parts of the Tana River area, have led to humanitarian and fiscal disasters (Opere 2013). The variability of extreme rainfall in the country has led to socio-economic challenges in urban and rural settings, including damage to infrastructure, loss of agricultural yields, and adverse effects on human health, e.g., the prevalence of water-related diseases (Juma, et al. 2020).

Kenya has also experienced a notable increase in severe and frequent drought events in recent decades. Drought events are predominantly experienced in the eastern and north-eastern regions of the country, as well as parts of the coast and Rift Valley—arid and semi-arid lands (ASAL) that form about 80% of Kenya’s land cover (Mutsotso et al. 2018; Owuor 2015). The occurrence of drought events in these regions has been exacerbated by the continuous decline in March–May (MAM) seasonal rainfall (Ayugi et al. 2020b). The communities in these areas, mainly pastoralists and agro-pastoralists, are made vulnerable by the adverse effects of the droughts, which include acute malnutrition, famine, increased conflict, interrupted food chains, and economic losses due to poor meat and milk production (Owuor 2015).

Research shows that over the past decade, developing countries, on the whole, have incurred over 35 billion USD a year in damages from natural disasters, 20 times the cost sustained in the developed world (Mirza 2003). There is growing concern regarding the increase in frequency and magnitude of these extreme weather events. These losses are expected to increase with time. These projections call for continuous assessment and monitoring of the evolving extreme weather patterns in the country, to develop effective adaptation measures to reduce risk and ensure that the region is prepared when unforeseen weather events occur (Kundzewicz, et al. 2014).

### Alternative data sources and identification of extreme weather events

In recent decades, the availability of new data sources, especially in areas like Kenya where both historical yield and weather data are not available, have supported endeavors in objectively identifying extreme weather events. Key among these resources are climate models, which have been employed to predict flood and drought weather patterns either by driving a hydrological model with meteorological data from a global or regional climate model (GCM or RCM) or employing hybrid statistical–dynamical techniques using projections as covariates within a statistical modelling framework (Addor et al. 2014; Hakala et al. 2019; Madadgar et al. 2016; Mendoza et al. 2015; Mizukami et al. 2016; Slater and Villarini 2018; Wilby 2010). Climate data from GCMs are of coarse space resolution and cannot represent fine-scale detail. Hohl et al. found that RCM outputs could provide more valuable insights into future climate variability and drought risk (Hohl et al. 2020). However, previous studies have found uncertainties with RCMs from boundary conditions, the size of the integration domain, and natural variability within the RCMs and RCM formulation (Luhunga et al. 2016; Meier et al. 2011; Min et al. 2013).

Machine learning techniques have been used for the classification and forecasting of extreme weather events using satellite and reanalysis data. Supervised machine learning classifiers such as logistic regressions, support vector machines, and neural networks have been used to identify drought, hurricanes, and tropical cyclones (Richman et al. 2016; Liu et al. 2016; Kim et al. 2019). In the absence of ground truth–labeled datasets, unsupervised anomaly detection methods (techniques for identifying unexpected events or patterns that differ from the norm in a given set) have been employed to identify extreme weather events including droughts and floods. Techniques such as density-based spatial clustering of applications with noise (DBSCAN), nearest neighborhood-based techniques have been used to identify extreme hot or cold temperatures, extreme weather patterns, and climate change (Çelik et al. 2011; Wibisono et al. 2021; Das and Parthasarathy 2009). In their review, Fung et al., Inoubli et al., and Mosavi et al. discuss the state of the art of artificial intelligence models used to predict drought indices and flood (Fung et al. 2020; Inoubli et al. 2020; Mosavi et al. 2018).

The objective of this study is to explore the potential of remote sensing datasets and determine whether machine learning models can improve the identification of extreme dry and wet weather events in selected regions in Kenya. First, we assessed different satellite datasets against ground-based rainfall data to evaluate how well these datasets best capture in situ measurements. Next, we assessed the performance of machine learning algorithms in predicting extreme weather events with respect to more traditional approaches.

## Methods

### Areas of study

**Fig. 1:**
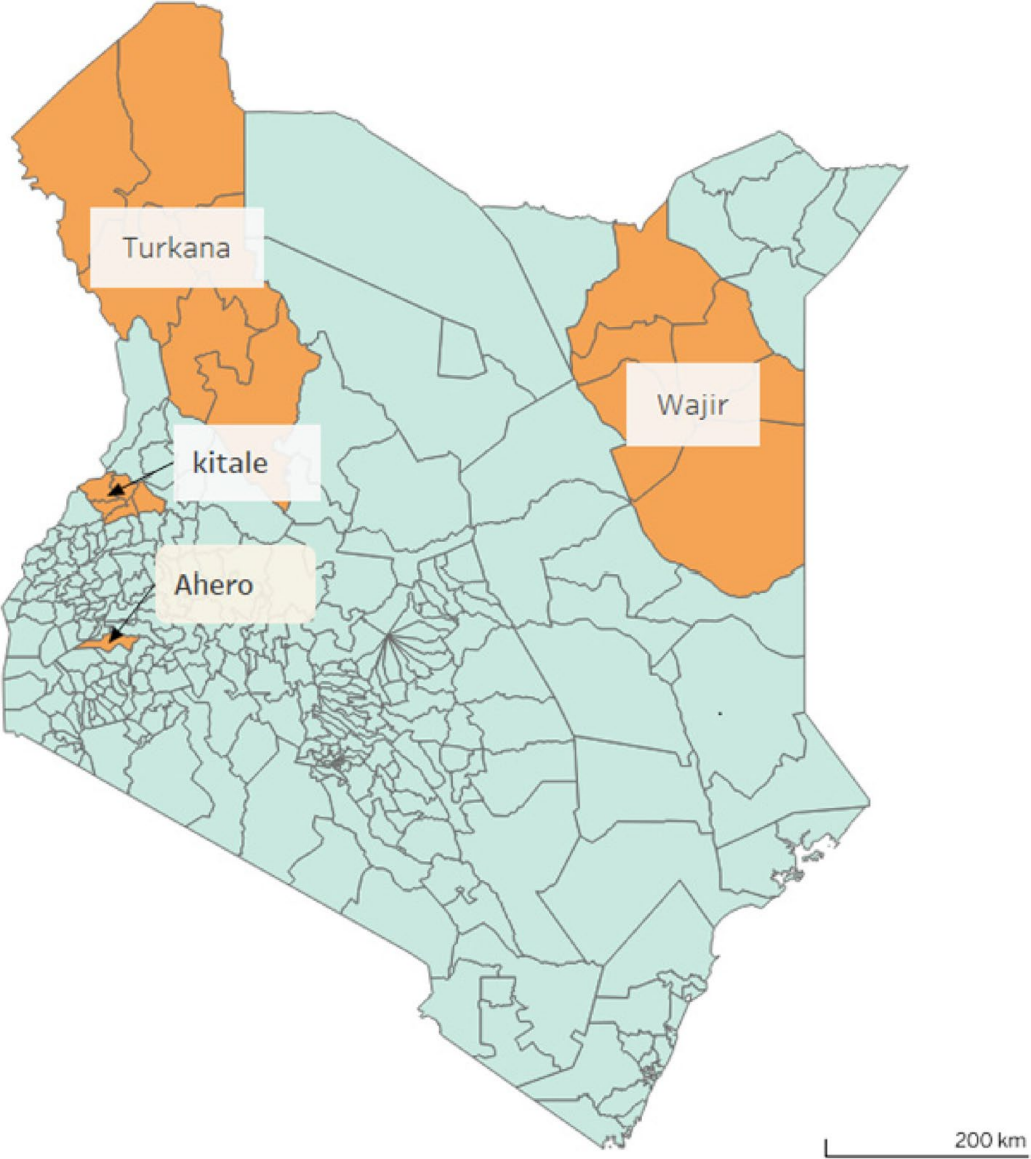
The image shows Ahero and Kitale towns the area of focus for the flood analysis and Turkana and Wajir counties in Northern Kenya, the areas of interest for the drought study. Basemap sourced from OCHA Regional Office for Southern and Eastern Africa (ROSEA) 2022)

The study focused on four regions in Kenya: Ahero town in Kisumu County and Kitale in northern Rift Valley for the flood analysis; Turkana and Wajir, located in Kenya’s most arid area, for the drought analysis as summarized in Fig. 1.

Ahero is located on the banks of River Nyando. It is commonly known for having low mean rainfall, approximately 7.72 mm. However, sporadic high torrential rainfall sometimes leads to pluvial floods in the area. Ahero is among the wards identified in Kenya with high exposure and vulnerability to flooding hazards (Makena et al. 2021).

Kitale is an agricultural town in northern Rift Valley Kenya situated between Mt. Elgon and the Cherengany Hills. It is situated at an elevation of 1900 m above sea level. It receives an annual rainfall of 1808 mm. Kitale is among the wards identified in Kenya with high exposure and vulnerability to flooding hazards; one notable instance of this is the 2008 flood which killed 24 people leaving 2396 affected (Huho and Kosonei 2014).

Turkana is located in the Northern region of Kenya. It has high-temperature levels that range between 36 and 38 °C. Wajir is located in the North-Eastern part of Kenya, where rainfall is scarce and there is low vegetation cover. According to the country’s National Drought Management Authority, both of these areas are prone to recurrent drought events (National Drought Management Authority 2020; National Management Authority 2020).

### Datasets

Conventionally, three types of datasets are utilized in climate modelling studies: observational products (satellite or gridded weather station), reanalysis products (obtained by combining observational inputs and climate models based on physical principles), and simulation products (climate models). This study used observational and climate datasets (the most commonly used datasets in data-sparse regions). Monthly precipitation data was obtained for both types of datasets for the period between 1992 and 2020.

Two satellite datasets were utilized: CHIRPS (Climate Hazards Group Infrared Precipitation with Station data) and TAMSAT (Tropical Applications of Meteorology using Satellite data and ground-based observations). CHIRPS is a quasi-global rainfall dataset that incorporates a 0.050 (4.4 km) resolution. It is mostly used in drought and flood monitoring. The University of Reading established TAMSAT to enhance the use of satellite-based rainfall estimates in Africa (Maidment et al. 2017). The satellite uses a 4 km resolution in the capturing of the rainfall data. Both satellite datasets have data spanning from 1992 to 2020 and are strictly for rainfall and therefore only utilized by the flood risk detection model.

#### Climate model data

Climatic data products, generally either GCM or RCM, are used in regions where accessing accurate and complete meteorological predictors at a high temporal resolution is difficult. At the regional level, however, GCMs fail to capture complex local processes and land-surface heterogeneities, which demand spatially detailed information at much finer scales than currently affordable with GCMs. Regional climate models (RCMs) are often used instead. RCMs use GCM outputs as boundary conditions and are dynamically downscaled to finer resolutions (e.g., 50 km) and consider mesoscale factors such as complex topography, coastlines, inland bodies of water, and land cover distribution over a limited region of interest at a computationally affordable cost (Luhunga et al. 2016; Meier, et al. 2011). Several RCMs of the Coordinated Regional Downscaling Experiment (CORDEX) Africa have been developed and used for analyses of projections of climatic conditions over East Africa. Three regional climate models (RCM) were selected from CORDEX (Coordinated Regional Downscaling Experiment) for the study:

- Max Planck Institute for Metrology Germany, MPI-M-MPI-ESM-LR (MPI)
- Canadian Centre for Climate Modelling and Analysis, CCCma-CanESM2 (CCCma)
- Met Office Hadley Centre, HAD-GEM2

The three RCMs were selected as they were found in previous studies to reproduce the rainfall climatology over the study domain with reasonable accuracy (Ayugi et al. 2020a; Luhunga et al. 2016). The three datasets were applied to the flood risk detection model, while the drought prediction models used the MPI and Had-GEM2.

#### Satellite data

Two satellite datasets were utilized: CHIRPS (Climate Hazards Group Infrared Precipitation with Station data) and TAMSAT (Tropical Applications of Meteorology using Satellite data and ground-based observations). CHIRPS is a quasi-global rainfall dataset that incorporates a 0.050 (4.4 km) resolution. It is mostly used in drought and flood monitoring. The University of Reading established TAMSAT to enhance the use of satellite-based rainfall estimates in Africa (Maidment, Black and Young, TAMSAT Daily Rainfall Estimates (Version 3.0). 2017). The satellite uses a 4 km resolution in the capturing of the rainfall data. Both satellite datasets have data spanning from 1992 to 2020 and are strictly for rainfall and therefore only utilized by the flood risk detection model.

#### Weather station data

Weather station data provides inferences for a single point compared to satellite or climate model datasets which capture a much larger area (TAMSAT and CHIRPS 4 km resolution and RCMs 50 km resolution). Weather station data was obtained from the Visual Crossing Weather Platform (https://www.visualcrossing.com), which provides daily historical forecasted data from any geographic location. The platform builds its historical weather data by combining raw surface observational data from various meteorological observational datasets such as the Integrated Surface Database (ISD) and the Meteorological Assimilation Data Ingest System (MADIS). The data acquired for this study spans the period from 1992 to 2020. Two additional datasets were also used: Trans-African Hydro-Meteorological Observatory (TAHMO), which was used to confirm the data bias of the visual crossing data, and World Weather Online data, which provides intra-day periods of data (3-h data up to 1-h data) for finer forecasts of the data. Weather station data have both temperature and rainfall data and are therefore applicable to the drought and flood risk models.

### Anomaly detection techniques and performance evaluation for flood risk detection

Four unsupervised anomaly detection techniques were utilized in the flood risk study: K-nearest neighbours (KNN), histogram-based outlier score (HBOS), cluster-based outlier factor (CBLOF), and isolation forests. These were used to determine the climate models’ ability to differentiate between extreme and non-extreme weather phenomena provided with specific threshold parameters. The threshold parameters were based on the national weather service standards of drought and flood risk weather events in the regions of interest.

K-nearest neighbours (KNN) is one of the simplest anomaly detection methods. It is a supervised machine learning algorithm that takes an unsupervised approach to anomaly detection. This density-based measure assumes that normal data points occur around a dense neighbourhood and anomalies lie far away. The algorithm performs a nearest neighbour search by computing the distances of every two data points then compares them to an arbitrary threshold value beyond which observations are identified as anomalies (Chandola et al. 2009). The main advantage of the KNN technique is that it is straightforward to adapt to different data types and only requires the definition of K and an appropriate distance measure for the given data. The study utilised a contamination rate of 10% with a K threshold of 5 and the Minkowski distance measure, a generalisation of the Euclidean and Manhattan distance measures.

Histogram-Based Outlier Score (HBOS) is a statistical-based anomaly detection technique (Goldstein and Dengel 2012). HBOS assumes that the data set features are independent and models the feature densities using normalised histograms (maximum height = 1) with a static or dynamic bin width. The height of every single bin of the histogram represents the density estimation. The outlier score for every data point is a multiplication of the inverse of the estimated densities, defined as:

1 $$HBOS\left(x\right)= \sum_{i=0}^{d}\mathrm{log}(\frac{1}{his{t}_{i}\left(x\right)})$$

Density estimation represented by a single bin in histogram-based outlier score (HBOS) calculation.

where $d$ represents the number of features in the data set, $his{t}_{i}(v)$ is the density estimation of each feature instance, and $x$ is the vector of features. Higher scores represent anomalies that would intuitively be assigned to bins with low density. This technique is fast compared to other techniques. For instance, it has a linear time complexity (*n*) compared to KNN’s (*n*^2^). HBOS is also a suitable option for treating global anomalies. However, it is a poor choice for local anomalies.

Cluster-based local outlier factor (CBLOF) is an anomaly detection technique that combines a nearest neighbour-based technique, local outlier factor (LOF), and clustering for data pre-processing and anomaly identification (He et al. 2003). The algorithm assigns each data point a CBLOF, which is determined by the distance of the data point to its nearest neighbour and the size of its cluster. Anomalies are identified as instances that belong to small or sparse clusters. CBLOF was designed to address shortcomings in clustering-based methods such as DBSCAN and nearest neighbour-based approaches such as Local Outlier Factor that were deemed ineffective. The technique is better at capturing local anomalies than HBOS. Additionally, its computational cost is minimised by distributing large data sets into meaningful clusters. To calculate the CBLOF, k-means is used to cluster the dataset. Then a heuristic procedure is applied to split the clusters into two categories based on their density. The anomaly score is defined as:

2 $$CBLOF\left(x\right)=\left\{\begin{array}{c}\left|C_i\right|^\ast\min\;\left(\mathrm{distance}\;\left(x,C_j\right)\right),\;if\;x\in C_i,\;C_i\in SC\;and\;C_j\in\;LC\;for\;j=1\;to\;b\\\left|C_i\right|^\ast\;\min\;\left(\mathrm{distance}\;\left(x,C_i\right)\right),\;if\;x\in C_{i\;}and\;C_i\in\;LC\end{array}\right.$$

Anomaly score in cluster-based local outlier factor (CBLOF) calculations.

where ${C}_{i}$ and ${C}_{j}$ represent clusters, LC = ${C}_{i} | i \le b$ represents large clusters, SC = ${C}_{j} | j>b$ represents small clusters, and $b$ is the cluster boundary (small or large).

Isolation Forests is an anomaly detection method for continuous data (Liu et al. 2012). The isolation forests technique makes two assumptions about anomaly data: anomalies vary significantly from normal observations making it easy to isolate, and anomaly data are rare in the dataset. Given these assumptions, the isolation of anomalies is implemented using an isolation tree ($i\mathrm{Tree}$), which is a binary tree structure that considers whether an observation is an anomaly or not. The algorithm recursively portions a dataset to build an ensemble of $i\mathrm{Trees}$. The anomaly score is derived from the path length, averaged over the isolation trees’ ensemble. Anomalies are identified as instances with short average path lengths on the $i\mathrm{Trees}$. Isolation forests have several advantages over other anomaly detection techniques. First, given its base assumptions, the algorithm can exploit subsampling, making it fast and scalable. Second, Isolation Forests can detect both clustered and scattered anomalies in the global context of the entire dataset. Isolation forests require two training parameters: the number of trees to build and sub-sampling size and one evaluation parameter: the tree height limit during evaluation. The anomaly score for every data point $x$ is defined as:

3 $$s\left(x,n\right)=2 \frac{E\left(h\left(x\right)\right)}{c\left(n\right)}$$

Anomaly score for individual data points in the isolation forest calculation.

where $E\left(h\left(x\right)\right)= \frac{{\sum }_{i=1}^{t}{h}_{i}\left(x\right)}{t}$ is the average path length of x over t $i\mathrm{Trees}$, $c\left(n\right)=2H\left(n-1\right)-\left(\frac{2\left(n-1\right)}{n}\right)$ with $H\left(i\right)=\mathrm{ln}\left(i\right)+ \gamma \left(\gamma is\mathrm{Euler}^{\prime}\mathrm{scontant}\right)$, the average path length of unsuccessful search in Binary Search Tree (BST). If $s\left(x, n\right)$ of $x$ is close to 1, $x$ is considered an anomaly or if less than 0.5, $x$ is considered normal. The isolation forest contamination rate for the study was set to 10% such that the confidence interval was at 90% for the flood risk detection rate to ensure high precision for anomaly identification.

Model performance was done by comparing model data with the Standardised Precipitation Index (SPI). The metric used here was the root mean square error to compare the ground truth SPI values with the ANN forecasted SPI values for the TAMSAT, CHIRPS, and GSOD weather datasets. SPI quantifies precipitation anomalies for long-term normal conditions on multiple time scales (McKee et al. 1993). Computation of the SPI involves fitting a probability distribution to an aggregated monthly precipitation time scale that may range from 3–48 months. The probability function is then transformed into a normal standardised index that is traditionally classified into flood risk classes that characterise the flood risk severity at each place and time scale. This study computed the SPI by fitting a logistic distribution for the different precipitation time series data. The data was converted to monthly intervals to maintain low granularity. SPI values greater than two were treated as the true observations of the occurrence of flood risk. Using the SPI values to label the occurrence of flood risk events, the study measured how well the anomaly detection models predicted the actual class of the data point using the following metrics:

- Fraction accuracy, which measured the fraction of the flood risk occurrences discovered by the outlier detection models over the number of flood risks the SPI found
- Specificity, which measured how well the model was able to determine when flood risk would not occur
  4 $$\frac{TN}{TN+FP}$$
  Model specificity formula.
- Sensitivity, which measured how well the model detected flood risk occurrences
  5 $$\frac{TP}{TP+FN}$$
  Model specificity formula.
  Note: TP is defined as the true positives from the model, TN is the model’s true negatives, FP the false positives and FN the false negatives from the model.
- AUC-ROC (AUC (area under the curve) and ROC (receiver operating characteristics)), which measured the ability of the anomaly detection methods to distinguish between flood risks and normal observations. The AUC-ROC curve is a performance measurement for classification problems at various threshold settings. ROC is a probability curve and AUC represents the degree or measure of separability.

### ANN model development and performance evaluation for extreme weather prediction

The ANNs were developed using the output from the Standardised Precipitation Evapotranspiration Index (SPEI) values for 28 years (1992–2020). SPEI is computed using precipitation and the potential evapotranspiration (PET) data to define anomalous wet and dry conditions by normalising the monthly (or weekly) difference between water supply (precipitation) and demand (potential evapotranspiration). This study calculated the PET following the Hargreaves equation (Hargreaves and Samani 1985). The Hargreaves equation uses the daily difference between the maximum and minimum as a proxy to estimate net radiation and simplifies the mass transfer term with a constant. Additionally, the study found the Hargreaves equation to be more suited to monthly durations.

6 $$PET=0.0023 \times {R}_{A}\times {\mathrm{TD}}^{0.5}\left(T{C}^{^\circ }+17.8\right)$$

Hargreaves equation for calculating potential evapotranspiration.

where ${R}_{A}$ measures the extra-terrestrial radiation calculated by knowing the station latitude, $TD$ is the mean maximum minus the mean minimum temperature, $TC$ is the mean temperature in degrees Celsius. The accumulated water profit or loss series, ${D}_{n}^{k}$, is computed using the following formula:

7 $${D}_{n}^{k}= \sum_{i=0}^{k-1}{P}_{n-i}-PE{T}_{n-i}, n\ge k$$

Accumulated water profit or loss formula.

where ${P}_{i}$ is the monthly precipitation, ${PET}_{i}$ is the monthly potential evapotranspiration, $k$ is the time scale in months, and $n$ is the calculation frequency. The D series was standardised using the log-logistic distribution based on the behaviour of the extreme values (Vicente-Serrano et al. 2010). A log transformation was performed to normalise the input values and control for extreme values in the data. The data was standardized using minimum maximum scaling to allow for comparison between the different weather datasets. The study converted the SPEI time series into binary data indicating whether a given period experienced drought or normal conditions.

We utilized a multi-layer perceptron (MLP), neural network model, along with a user-created neural network. The selected ANNs used a two-layer neural network with four input layers, eight hidden layers, and one output layer. The models used feed-forward backpropagation (BPN) as the training algorithm. The BPN utilised a sigmoid activation function since the output is of a binary form. A learning rate of 0.0001 was used to maximise the creation of the best log loss slope—for all the models, the same parameters were put in place to see how they all differ.

The data was split into a train-test and validation set up for each data set, where 90% of the data was used for training and testing (80:20). The remaining 10% was used in the validation. The training data consisted of the minimum and maximum temperature and precipitation variables. The SPEI value grading for drought analysis was capped at < − 1.5 to signal the start of drought occurrences.

The performance of the ANN models in predicting the monthly SPEI was statistically evaluated using the accuracy score and the AUC-ROC.

- The accuracy score measures the fraction of correct predictions out of the total number of observations.
  8 $$\mathrm{Accuracy}= \frac{TP+TN}{TP+FP+FN+TN}$$
  Model accuracy formula.
- The AUC-ROC measures the models’ capacity to distinguish between drought and normal (non-drought rainfall measures) observations.

## Results

### Flood Risk Detection in Ahero County in Western Kenya

Four anomaly detection models (CBLOF, HBOD, KNN, IF) and six datasets (CHIRPS, MPI, Had-GEM2, TAMSAT, CCCma, Weather Station Data) were analysed to determine their accuracy in detecting flood risks in Ahero county. Table 1 summarises descriptive statistics of precipitation data obtained from the selected open-source observational and simulation datasets. The datasets varied greatly in terms of precipitation distribution. TAMSAT data recorded the highest mean precipitation of 3.84 mm (± 0.79), while the weather station data had the highest maximum precipitation recorded over the study period, 236 mm. The RCMs recorded the lowest precipitation over the period compared to the observational products. The Met Office Hadley Centre Regional Climate Model (Had-GEM2) had the lowest average rainfall of 0.57 mm (± 2.43). The differences between observed and simulated annual rainfall have been attributed to the poor skill of RCMs in reproducing the regional climate over East Africa (Ayugi et al. 2020a; Bichet, et al. 2020; Mumo and Yu 2020).

**Table 1:** A comparison of precipitation data for Ahero county across the 6 different datasets analyzed

	MPI	CCCMA	HAD-GEM2	CHIRPS	TAMSAT	Weather station
Number of observations	10,593	10,593	10,593	10,593	10,593	10,573
Mean precipitation	0.95	0.65	0.57	0.79	3.84	1.13
SD precipitation	3.48	2.7	2.43	4.10	4.79	8.44
Min precipitation	0.00	0.00	0.00	0.00	0.00	0.00
Max precipitation	48	34	35	70.07	35.90	236.00

**Fig. 2:**
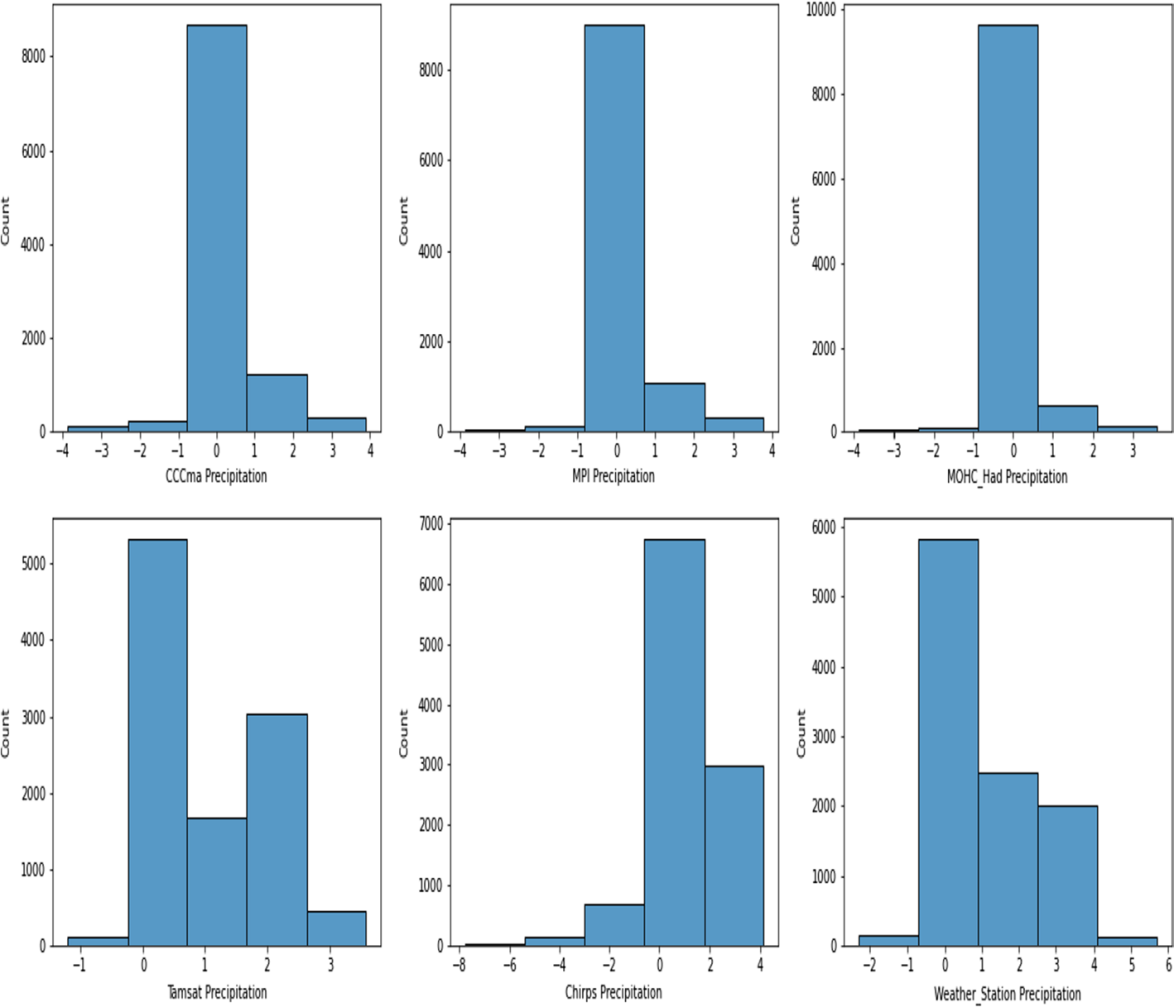
Distribution of precipitation in Ahero across the selected datasets: CCCma, MPI, Had-GEM2 TAMSAT, CHIRPS, and Weather Station Data

Figure 2 illustrates the precipitation distribution in Ahero over the observation period and highlights the differences in precipitation data across the datasets. Precipitation over the region features a positive skew, with few instances of extreme rainfall. This distribution is carried across all datasets. However, we find that the precipitation data obtained from RCMs recorded no or low precipitation in the region over the observation period compared to the other datasets. Previous evaluations of RCM performance over the East African region found RCMs underestimate rainfall, especially during the March–April-May (MAM) seasons (Luhunga et al. 2016). Previous studies report the poor performance of RCMs of the region in reproducing rainfall events when compared to observational data. This is attributed to various factors, including their different abilities to simulate mechanisms behind rainfall formation over the region due to the paucity of in situ data required for model parameterization (Mumo and Yu 2020).

The number of flood risks identified by the SPI varied with each dataset. The variation in the number of flood risks across the selected datasets is due to the differing mechanisms in which the data products estimate rainfall. Each model performance was assessed against the unique characteristics of the data used; model performance varied with each dataset. In general, the anomaly detection models were able to identify more than half of the flood risk anomalies in the CHIRPS dataset but performed poorly in identifying flood risk anomalies in the climate model datasets. The best performance was obtained by the CBLOF anomaly detection method using the CHIRPS dataset. Eighty percent of the anomalies detected had been identified as flood risk events based on the SPI. Precipitation distribution in the RCM datasets may be the reason for their poor performance in flood risk detection; most of the observations in the RCM data had no or low precipitation values recorded. Table 2 details the number of flood risk anomalies detected by each model as a fraction of the number of flood risks identified by the SPI (last row):

**Table 2:** Algorithm performance calculated as a function of the number of flood risks detected compared to the number identified by SPI

	CHIRPS	Weather station	TAMSAT	CCCMA	MPI	HAD-GEM2
CBLOF	80%	34%	25%	5%	1%	0%
HBOD	67%	46%	-	5%	1%	0%
IF	67%	33%	20%	1%	1%	0%
KNN	67%	40%	50%	5%	1%	-
No. floods SPI	30	19	17	28	23	24

Model specificity was determined for each type of dataset. All models performed well in terms of classifying non-flood risk events correctly when using weather station data but were less accurate when utilizing climate model data. Table 3 summarizes the model specificity (the values indicate the ability of the model to identify non-flood risk events):

**Table 3:** Model specificity in classifying non-flood risk events

	Weather station	CHIRPS	TAMSAT	CCCMA	HAD-GEM2	MPI
HBOD	1	0.17	0	0	0	0
CBLOF	1	0.14	0	0	0	0
IF	1	0.17	0	0	0	0
KNN	1	0.25	0	0	0	0

Additionally, model sensitivity was determined against each type of dataset. All four models were able to identify flood risk anomalies correctly for each of the six dataset types (Table 4). However, this may be attributed to the zero-inflation (distribution that allows for frequent zero-valued observations) of the TAMSAT and the climate model data, indicating how skewed these datasets are in the detection of flood risks.

**Table 4:** Model sensitivity that measures the model ability to identify flood risk anomalies

	CHIRPS	Weather station	TAMSAT	CCCMA	HAD-GEM2	MPI
HBOD	0.97	1	1	1	1	1
CBLOF	0.96	1	1	1	1	1
IF	0.97	1	1	1	1	1
KNN	0.944	0	1	1	1	1

Finally, Table 5 summarizes the area under the curve receiver operating characteristic (AUC-ROC) performance measure, which provides information on the models’ ability to differentiate between flood and non-flood risk events. The isolation forest model using weather station data had the best performance in distinguishing between flood risk events with an AUC-ROC measure of 0.8. Unlike HBOD, CBLOF and KNN, IF models identify anomalies independent of the underlying data distribution; the other anomaly detection algorithms build a profile of normal instances based on distance or density; high-density regions or short distances measured in a local context could be anomalies in the global data set or vice versa. Isolation forest path-length-based isolation traverse data to identify anomalies in local and global contexts. This may account for the discrepancies in performance between the 4 models. The CBLOF model also had good performance with the same dataset. The models performed especially poorly using data from the regional climate models. All models could not differentiate between flood and non-flood risk events using RCM data. The poor performance may be attributed to the precipitation distribution, which underestimated rainfall in the region, leading to an inflation of zeros in the dataset.

**Table 5:** Algorithm performance in differentiating between flood and non-flood risk events as ascertained using the AUC- ROC performance measure

	CHIRPS	Weather station	TAMSAT	CCCMA	HAD-GEM2	MPI
HBOD	0.57	0.5	0.5	0.5	0.5	0.5
CBLOF	0.55	0.732	0.5	0.5	0.5	0.5
IF	0.566	0.8	0.5	0.5	0.5	0.5
KNN	0.597	0.5	0.5	0.5	0.5	0.5

### Drought detection in Turkana and Wajir counties in Northern Kenya

#### Drought detection using SPEI

**Fig. 3:**
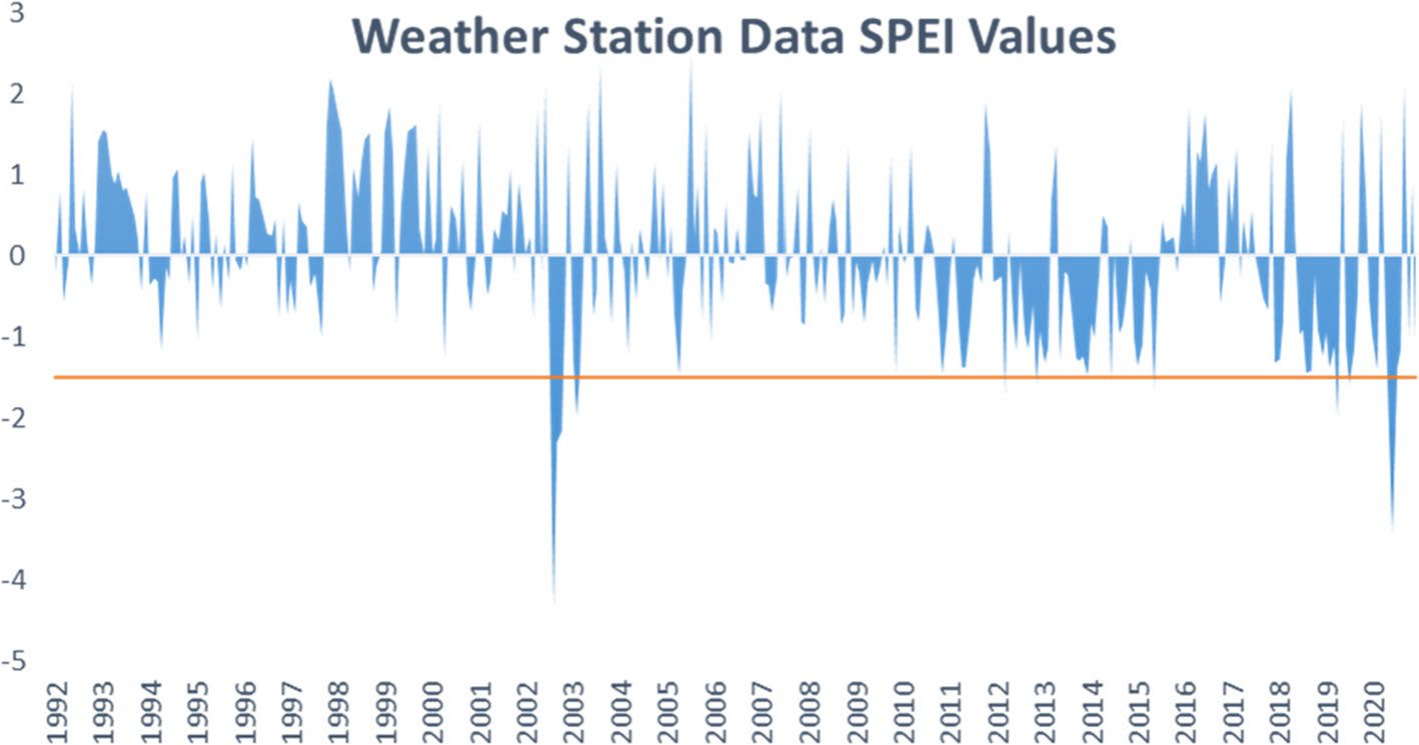
One-month SPEI variability in Weather Station Data for the years 1992–2020

**Fig. 4:**
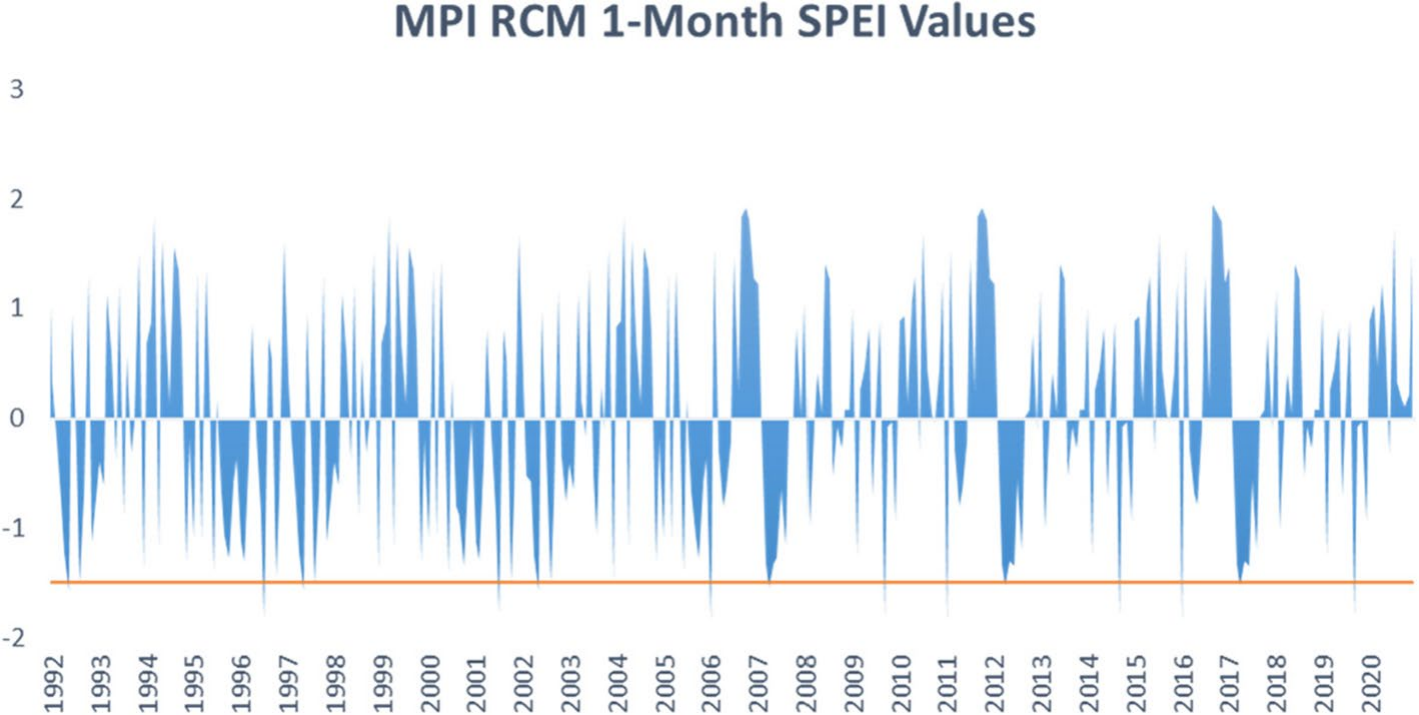
One-month SPEI variability in MPI RCM for the years 1992–2020

**Fig. 5:**
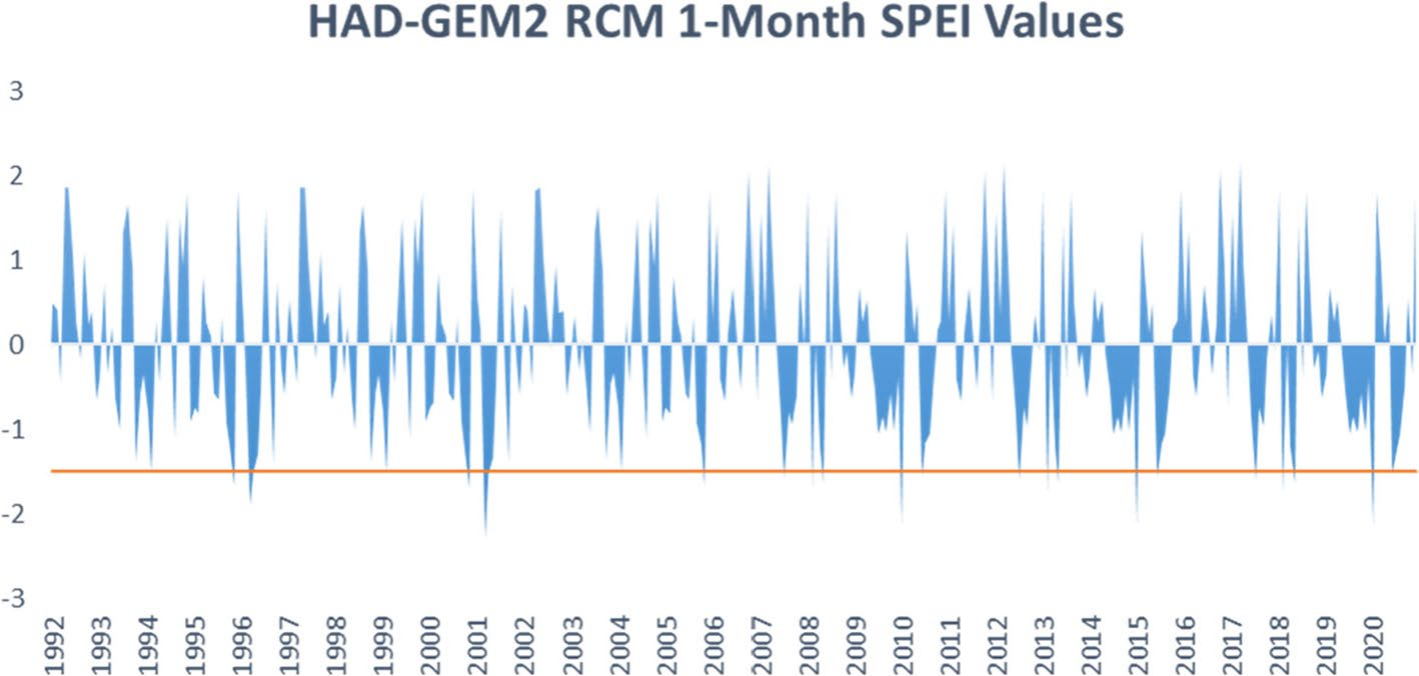
One-month SPEI variability in Had-GEM2 RCM for the years 1992–2020

SPEI was computed for the selected region using the three datasets. Figs. 3, 4, and 5 summarize the 1-month SPEI variability in the three datasets for the period between 1992 and 2020 in Turkana and Wajir counties. Positive SPEI values indicate greater than normal precipitation, while negative values indicate less than normal precipitation. Therefore, extreme peaks and troughs in the graphs indicate flood risk or drought events, respectively. A cutoff point of − 1.5 was used to identify extreme drought events in the two regions in the study. There is evidence of within-season variability with experiences of moderate to severe and moderate to extreme drought cases within the period. Examining Fig. 2, 1-month SPEI values computed from weather station data show dry events in the region occur towards the end of 2002 and early 2003; another long dry event starts in 2012 and ends in 2015 with intermittent wet periods, and again in the period from 2019 to 2020. Extreme drought events occurred in 2012 and the start of 2020.

The MPI and Had-GEM2 RCMs capture several instances of drought events as summarized in Figs. 3 and 4, respectively. Moderate drought events were observed during the period of 1992, 1996, 1997, 2001, 2002, 2007, 2009, 2011, 2012, 2014, 2016, 2017, and 2019 for MPI. While the Had-GEM2 captured extreme drought events in 2001, 2010, 2015, and 2020 in addition to the moderate events captured by the MPI dataset.

The droughts captured in the observational data and the regional climate models correspond to drought incidences reported in the literature (Ayugi et al. 2020a, b; Owuor 2015). However, the time and frequency of drought instances differed between the datasets. The discrepancies can again be attributed to varying precipitation distribution between the observed weather station data and simulated RCM datasets.

#### ANN drought forecasting model

Different ANNs were tested for forecasting the SPEI using weather station data and climatic model data. Table 6 lists the accuracy scores for the training and validation period. There is some reduction in the evaluation metric during the validation period. The low values in the validation period indicate that there is possibly some zero inflation, where the model predicts one scenario overall. Weather station data using the MLP neural network gave the best performance for 1-month drought forecasting. It had the highest training and validation accuracy scores and the lowest reduction in accuracy scores between training and validation. Among the regional climate models, Met Office Hadley Centre, Had-GEM2 provided the best validation accuracy at 57%.

**Table 6:** Accuracy scores of ANNs for 1-month drought forecasting of SPEI

	Weather station data		MPI		HAD-GEM2	
	Training	Validation	Training	Validation	Training	Validation
User-created neural network	90%	48%	87%	45%	81%	57%
Multi-layer perceptron (mlp) neural network	78%	63%	63%	54%	70%	57%
Validation accuracy	73%		63%		40.8%	

ANN models using weather station data had the highest AUC-ROC scores—indicating that the anomaly detection models could adequately distinguish between drought and non-drought events (Table 7). The regional climate models had low scores in this respect except for the HAD—GEM2 data using the MLP neural network. Additionally, AUC-ROC increased to 0.65 in the validation period for weather station data, indicating the model’s generalisability. The regional climate models, MPI and HAD-GEM2, had much lower scores in this respect, with the exception of the HAD-GEM2 data using the MLP neural network. MLP performance was superior to the user-generated neural network due to the use of an Adam optimiser. The discrepancies between the RCM and Weather Station data results are difficult to attribute to a specific source. RCMs have deficiencies in projecting the observable climate in the East African region, but weather station data accuracy is also limited (generally, by the number of weather stations used for interpolation, incompleteness, or inaccuracies of station records).

**Table 7:** AUC-ROC scores for the ANNs

	Weather station data	MPI	HAD-GEM2
User-created neural network	0.79	0.48	0.44
Multi-layer perceptron neural network (MLP)	0.6	0.45	0.62

It is worth noting that model and dataset performance could only be evaluated against SPEI values as there are no drought or flood repositories with historical weather data on these phenomena in Kenya. Ground truth data is needed to validate the above findings.

### Summary statistics for Kitale and Wajir precipitation data in Kitale town in Northern Rift Valley Kenya

For Kitale 444 data points were obtained after resampling the daily rainfall data to monthly rainfall data. CHIRPS recorded the highest average monthly rainfall of 108 mm followed by TAMSAT at 92.5 mm and lastly by GSOD at 39.82 mm. For the maximum precipitation, GSOD had the highest which was 1998.8 mm, followed by CHIRPS which was 386.5 mm then lastly by TAMSAT at 293.8 mm. The lowest rainfall recorded was 0 mm across all datasets. As for variation between data points, GSOD had the highest with a standard deviation of 170.58 mm followed by CHIRPS with 74.06 mm, and lastly by TAMSAT with 62.29 mm (Table 8).

**Table 8:** A comparison of the summary statistics of Kitale precipitation according to the three precipitation datasets analysed

	GSOD precipitation	CHIRPS precipitation	TAMSAT precipitation
Number of observations	444	444	444
Average rainfall	39.82	108.41	92.55
Standard deviation	170.58	74.06	62.29
Minimum rainfall	0	0	0
Maximum rainfall	1999.8	386.5	293.8

For Wajir 444 data points were obtained after resampling the daily rainfall data to monthly rainfall data. CHIRPS recorded the highest average monthly rainfall of 28.34 mm followed by TAMSAT at 24.73 mm and lastly by GSOD at 10.04 mm. For the maximum precipitation, CHIRPS had the highest which was 293.30 mm, followed by TAMSAT which was 265.45 mm then lastly by GSOD at 205.53 mm. The lowest rainfall recorded was 0 mm across all datasets. As for variation between data points, CHIRPS had the highest with a standard deviation of 48.51 mm followed by TAMSAT with 42.71 mm and lastly by GSOD with 32.59 mm (Table 9).

**Table 9:** A comparison of the summary statistics of Wajir precipitation according to the three precipitation datasets analysed

	GSOD precipitation	CHIRPS precipitation	TAMSAT precipitation
Number of observations	444	444	444
Mean rainfall	10.04	28.34	24.73
Standard deviation	32.59	48.51	42.71
Minimum rainfall	0.00	0.00	0.00
Maximum rainfall	205.53	293.30	265.45

#### RMSE and correlation analysis between the three datasets for Kitale and Wajir

##### Kitale

This study used the RMSE and correlation analysis to compare the daily TAMSAT and CHIRPS rainfall data with the daily ground truth GSOD data. For Kitale TAMSAT had an RMSE of 12.18 mm while CHIRPS had an RMSE of 14.06 mm. For the correlation analysis TAMSAT had a 0.055 correlation with GSOD while CHIRPS had a 0.023 correlation with GSOD. From this TAMSAT is the closest to the ground truth based on the two metrics.

##### Wajir

For Wajir TAMSAT had an RMSE of 7.53 mm while CHIRPS had an RMSE of 7.39 mm. For the correlation analysis TAMSAT had a 0.049 correlation with GSOD while CHIRPS had a 0.043 correlation with GSOD. From this TAMSAT is the closest to the ground truth based on the two metrics.

##### Heavy Rainfall Forecasting in Kitale town in Northern Rift Valley Kenya

In running the GSOD data through the SPI, only 9 occurrences met the criteria of heavy rainfall instances. However, on forecasting the SPI for TAMSAT and CHIRPS dataset using the MLP, an RMSE of 1.38 and 1.55 was obtained respectively. Comparing the CHIRPS GEFS SPI values to the forecasted TAMSAT and CHIRPS SPI values the obtained RMSE was 1.

### Drought forecasting in Wajir County in Northern Kenya

**Fig. 6:**
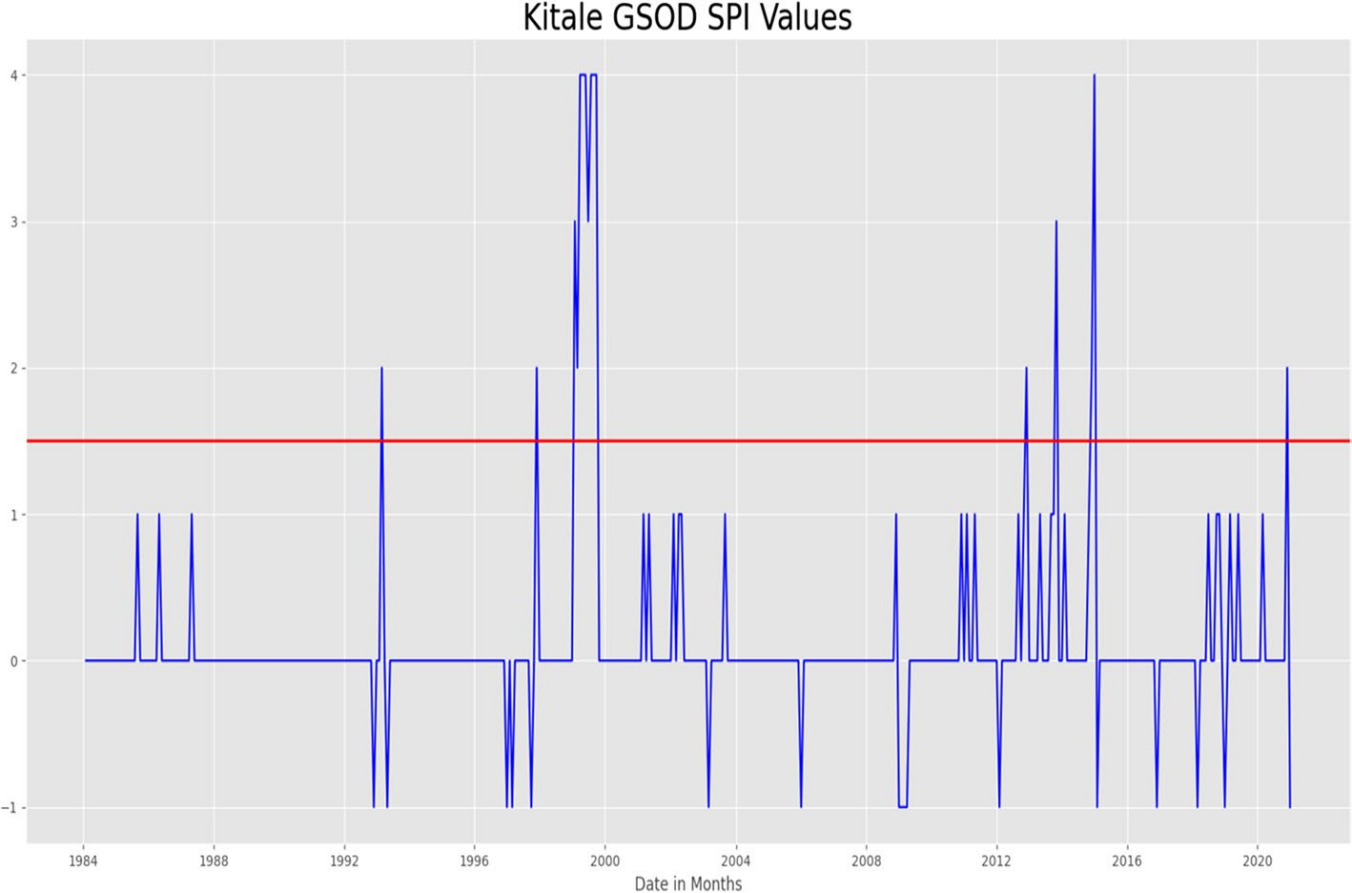
SPI values meeting the heavy rainfall threshold

**Fig. 7:**
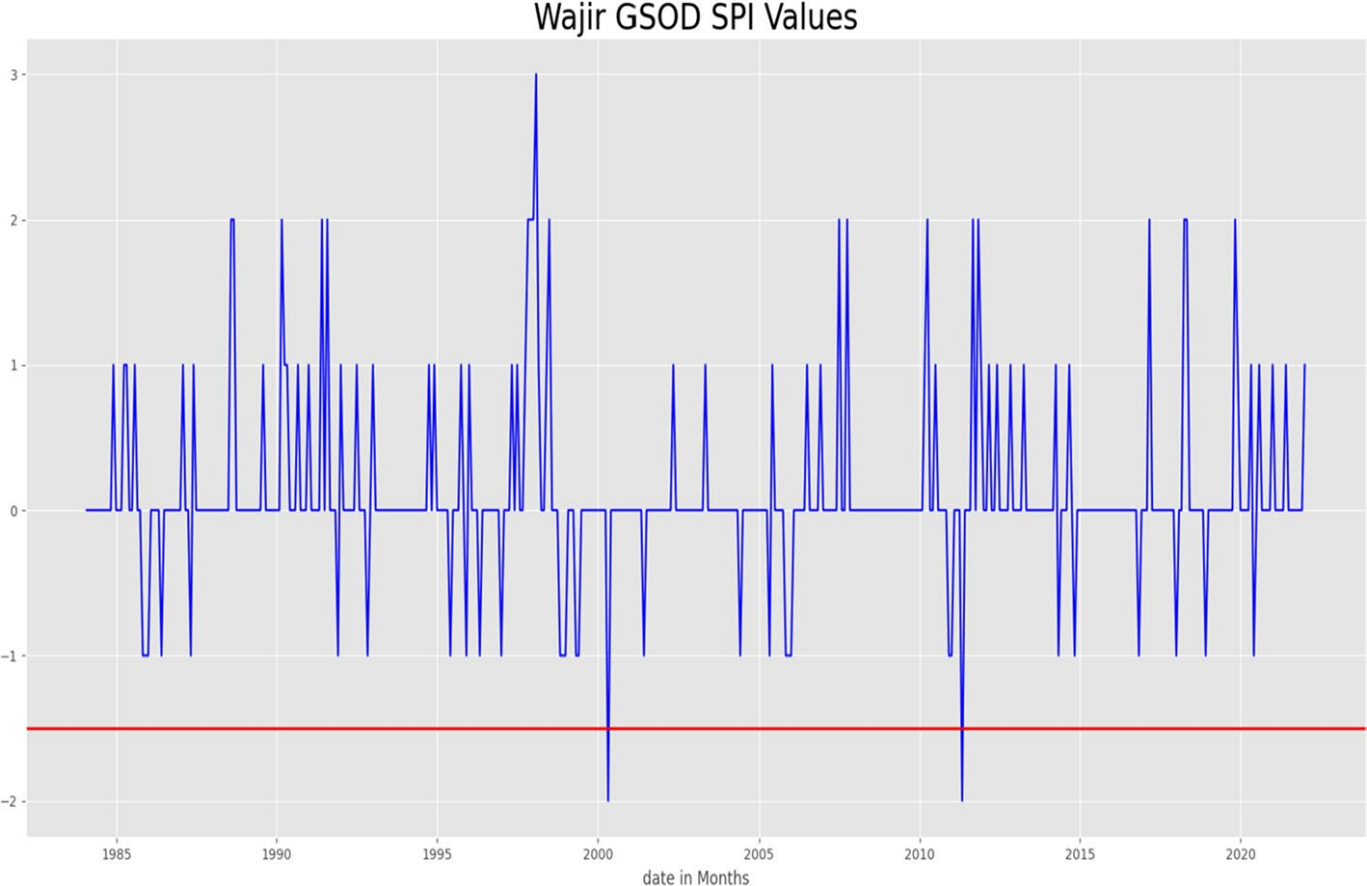
SPI values for Wajir meeting drought criteria

On running the SPI on the GSOD ground data for the whole dataset, the SPI classified two instances of precipitation as drought (Figs. 6 and 7). On fitting the Multilayer Perceptron Neural Network and forecasting the SPI values for both CHIRPS and TAMSAT, the MLP generated an RMSE of 0.79 for TAMSAT data, and 0.82 for CHIRPS. Comparing the CHIRPS GEFS SPI values to the forecasted TAMSAT and CHIRPS SPI values the obtained RMSE was 1.2

## Discussion and conclusion

The main resource for agricultural production, the land, is heavily affected by agro-climatic conditions—meteorological events heavily influence crop farming and animal husbandry outcomes. Thus, extreme weather events often adversely impact farmers and pastoralists; and contribute to rural poverty. Farmers can lose their crops, means of production, and even access to their land through extreme weather events like flood risks and droughts. An increase in the intensity and frequency of these extreme weather events due to climate change will result in a corresponding increase in the negative impacts on agricultural productivity. Accurately determining the occurrence, and intensity, of future drought and flood risk events could be used as an effective early warning system to prevent significant crop and animal loss and to develop mitigating strategies.

In this study, we identified two models that may be used to predict flood risk and drought events. The combination of ANNs and weather station data was the most effective in predicting future drought occurrences in Turkana and Wajir counties (both drought-prone regions) with accuracies ranging from 78 to 90%. In the case of flood risk forecasting, Isolation Forests models using weather station data had the best overall performance. The study also found that the regional climatic models (Max Planck Institute for Metrology Germany, MPI-M-MPI-ESM-LR (MPI; Canadian Centre for Climate Modelling and Analysis, CCCma-CanESM2 (CCCma), and Met Office Hadley Centre, HAD-GEM2 utilized struggled to identify flood risk anomalies in Ahero county (all three model performance of monthly aggregation was very low) but the RCMs from CORDEX-Africa (Max Planck Institute for Metrology Germany, MPI-M-MPI-ESM-LR (MPI) and Met Office Hadley Centre, HAD-GEM2 utilised in the study had adequate performance in drought forecasting. Superior weather station data performance, in both flood risk and drought forecasting, could be attributed to the singe-point measurements provided by weather station data which are usually more accurate when compared to reanalysis or simulation data products. This suggests that improvements in coverage of weather station data in data-sparse regions would increase the accuracy in drought and flood risk forecasting of climate models which would, in turn, increase accuracy in the underwriting process.

The use of ANNs on the CHIRPS and TAMSAT datasets for Kitale and Wajir townS provided relatively accurate heavy rainfall and drought forecasts respectively given their low RMSEs. The ANN SPI forecast has an RMSE of 1.21 and 0.66 with Wajir’s CHIRPS and TAMSAT data while for Kitale the ANN SPI forecasts for CHIRPS and TAMSAT data are 1.29 and 1.04 respectively when compared to the CHIRPS GEFS. Given the high forecasting performance exhibited by CHIRPS and TAMSAT data for drought and heavy rainfall, even though there are only a small number of ground weather stations in Kenya, shows that TAMSAT and CHIRPS can contribute towards data creation to complement limited ground weather data.

Future work, in the second phase of this project, will develop a model that utilizes the two identified drought and flood predictive climate change models, ANN and Isolated Forest models with weather station data, respectively, to calculate the risks of crops grown in the regions of the study mentioned in this paper. This model will be analysed against current methods of calculating risk for agricultural insurance policies in Kenya to determine which process is more efficacious.

## Data Availability

The datasets generated and analysed during the current study are available on the Zenodo platform, https://doi.org/10.5281/zenodo.5730074 (https://zenodo.org/record/5730074#.YgOeSN9BzIU).
